# A comparison of the efficacy of the effect of online versus face-to-face group counseling based on positive-approach on sexual intimacy of women after benign abdominal hysterectomy: a clinical trial

**DOI:** 10.61622/rbgo/2024rbgo102

**Published:** 2025-01-23

**Authors:** Orly Nazanin Kalimi, Tayebeh Mokhtari Sorkhani, Ali Dehghani, Mahshid Bokaie

**Affiliations:** 1 Shahid Sadoughi University of Medical Sciences Yazd Iran Shahid Sadoughi University of Medical Sciences, Yazd, Iran.

**Keywords:** Counseling, Feminility, Hysterectomy, Sexual behavior, Surveys and questionnaires

## Abstract

**Objective:**

The study investigates the influence of positive-approach counseling through both online and face-to-face group therapy on the sexual intimacy of women after benign complete abdominal hysterectomy, addressing challenges such as the loss of femininity and other psychosexual complications that disrupt the couple’s relationship post-surgery.

**Methods:**

This is a parallel clinical trial, conducted in 2023 in Yazd, Iran; with sixty-six participants post- benign complete abdominal hysterectomy were randomly assigned to online and face-to-face counseling groups. Each group had eight 90-minute sessions, and data were collected using demographic and intimacy scale (IS) questionnaires at baseline, eighth week, and twelfth week follow-up. Statistical analysis used SPSS version 23 (P < 0.05).

**Results:**

In the Online Group, the mean sexual intimacy score significantly increased from 72.42 ± 9.05 to 87.06 ± 7.98 at eight weeks and 90.30 ± 8.23 at twelve weeks (P < 0.001). In the Face-to-Face Group, the mean score increased from 70.21 ± 6.75 to 81.24 ± 5.55 at eight weeks and 85.03 ± 5.40 at twelve weeks (P < 0.001). Online counseling proved more effective than face-to-face counseling in enhancing sexual intimacy (P = 0.043).

**Conclusion:**

Online and face-to-face counseling based on the positive approach improved sexual intimacy in women with a history of benign hysterectomy. Moreover, it seems that online counseling was more effective, so it is recommended that this method be employed in follow-up sessions after hysterectomy. Iranian Registry of Clinical Trials - IRCT20230209057373N1

## Introduction

Gynecological complications cause much concern among women and affect every aspect of their lives.^(1)^ Currently, uterine diseases and disorders put women’s health at much risk.^(2)^ Becoming aware of uterine complications and infertility makes women’s sexual identity and behavior susceptible to feelings of insufficiency and not being able to control anger, guilt, and shame, and also causes problems in their relationships.^(3)^ One of the most common treatments for uterine diseases is hysterectomy.^(2)^

Hysterectomy is the most common gynecological operation in England and USA, and thirty-two percent of women in the Netherlands experience it in their lives.^(4)^ The WHO reported that in 2002, due to various complications, 146,422 hysterectomies were performed in Iran.^(5)^ Hysterectomy is mainly performed for benign uterine lesions in women and is one of the most common surgeries in women with benign uterine disease.^(6)^ Among benign uterine complications, abnormal uterine bleeding, adenomyosis, uterine prolapse, uterine myomas, endometriosis, and pelvic inflammatory disease are the most common causes of hysterectomy.^(7)^ Post-hysterectomy sexual dysfunction and disorder are reported in 10% to 40% of the cases.^(4)^

The more a couple are sexually attracted to each other, the more satisfaction and intimacy they enjoy in their sexual relationship. Thus, hysterectomy-induced feelings of loss of femininity and other psychosexual complications disrupt the couple’s sexual intimacy and relationship.^(8)^ Bagarozzi^(9)^ defines marital intimacy as closeness, similarity, and personal romantic or emotional relationship, which requires mutual understanding and appreciation.

Sexual intimacy includes mutual support and deep appreciation of each other to be able to express thoughts and feelings, connect, and share activities, and requires a level of commitment and affection to maintain the relationship.^(10)^ Sexual intimacy is sharing romantic feelings, desiring physical contact (embracing, kissing, and lovemaking), sexual fantasies, and intimate moves that are planned to arouse and achieve sexual satisfaction.^(9,10)^Sexual intimacy is deeply related to a couple’s quality of life, affects their sexual compatibility and mental health,^(11)^ and acts as a remedy for daily stress in their relationship.^(12)^Besides, lack of sexual intimacy is one of the most common causes of breakups and disruptions in relationships.^(13,14)^Since sexual intimacy and dysfunction are related, lack of intimacy indicates sexual dysfunction and emotional problems to some extent.^(15)^ It has been demonstrated that post-hysterectomy vaginal dryness and reduced sexual desire dramatically decrease sexual satisfaction.^(16)^There are many psychological approaches like relationship therapy, psychoanalytical approach, cognitive behavioral approach, educational psychology, and emotional psychology to treat marital problems such as lack of sexual intimacy and each approach has its own therapeutic and educational merits to improve sexual intimacy based on their special characteristics.^(17)^ Positive psychology is a new approach that pursues a pleasant picture of life and to determine what makes life valuable, it uses objective psychological methods.^(18)^The main subject of positive psychology is to investigate positive mental experiences. Wellbeing, happiness, satisfaction, pleasure, hope, optimism, dignity, and love are examples of such experiences.^(19)^ In teaching positive thinking, the individual is encouraged to appreciate their own and other’s potential abilities and advantages, recognize their role in elevating their self-respect and well-being, and achieve the ability to identify positive aspects.^(20)^ It is a suitable approach for treating women with marital problems.^(18)^

Nowadays, with technological advancements and transformation in counseling, the use of the Internet in counseling is considered an innovative alternative,^(21)^ so using the Internet to deliver health care is growing day by day.^(22)^Online counseling has benefits for patients with sexual problems because they maintain their anonymity and experience less stress and tension.^(15)^As patients can receive therapy without any concern about the location, it can be the first stage of treatment for patients with sexual problems.^(23)^The goal of online counseling is to provide an exchange of information between therapist and client and improve the patient’s behavior and mental health.^(24)^It has been concluded that online intervention has improved sexual intimacy and function, emotional intimacy, and communication.^(25)^According to previous study results and considering new studies confirming the effectiveness of online therapy in addressing a wide range of conditions related to midwifery and gynecology,^(26)^ sexual intimacy can improve commitment and relationships.^(27)^ Therefore, this study was conducted to compare the effectiveness of online and face-to-face counseling in patients after benign hysterectomy.

## Methods

This clinical trial with a parallel design was conducted from May to July 2023 on women who had undergone Benign complete abdominal hysterectomy. The research site was Shohada Kargar Hospital, Yazd, Iran.

Among the participants who had been referred to Shohada Kargar Hospital, Yazd, the information of 91 hysterectomy patients was checked, and 80 participants with benign hysterectomy were invited to participate in the study by phone. We obtained the informed written agreement of participants to observe the principles of ethics in the research. In the end, 66 participants who met the inclusion criteria were included in the study. Based on Mohammadi-Zarghan and Ahmadi’s study,^(28)^ taking into account the significance level of 0.05, type II error level of 0.02, power of the test of β-1 = 0.80, and 10% drop-out, 66 people were invited to the study.


$$n=\frac{2s^{2}p{\left[z_{1-a/2}+z_{1-\emptyset }\right]}^{2}}{\mu^{2}d}$$

**Figure 1:**
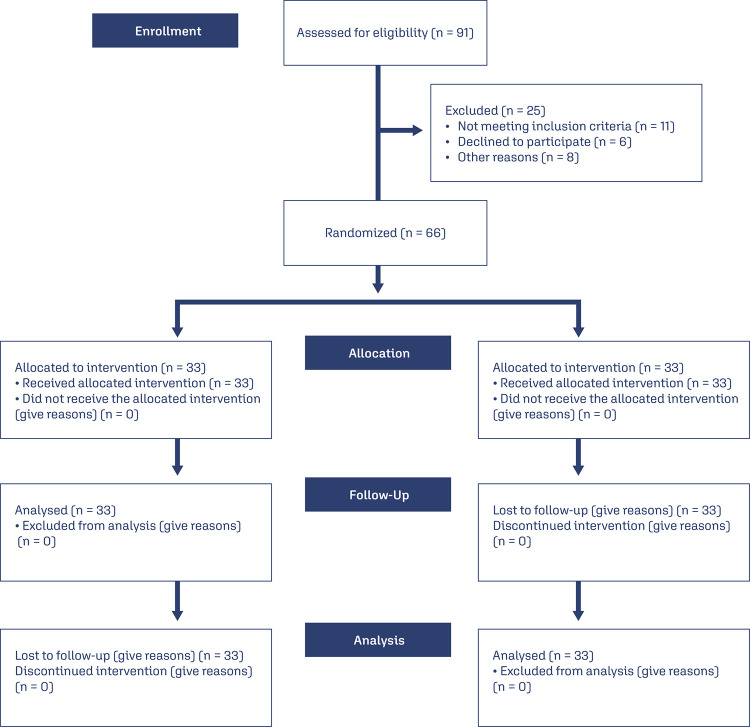
Flowchart illustrating study participation

After considering the inclusion and exclusion criteria, 66 participants were entered into this study. After using online software (www.Random.org/sequences), the participants were divided into two groups (33 women in online counseling and 33 in face-to-face counseling) (Figure 1). The concealment was done by the center’s receptionist who was outside the research team.

Inclusion criteria were; Age between 20–60 years, Cause of hysterectomy (benign), Type of hysterectomy (complete abdominal), Time since the hysterectomy (three months to two years), Shared bed with the spouse, The only wife of the spouse, Access to a smartphone and the Internet. Exclusion criteria were; Simultaneous participation in other psychological and psychiatric interventions, Taking psychiatric drugs (according to the patient’s history), Vaginal hysterectomy, and Drug abuse (according to the patient’s history). In case of absence in more than two counseling sessions and unwillingness to continue cooperation with the research team, the individual was excluded from the data analysis. In this study, due to the explanation of the objectives of the study, there was no dropout. Demographic questionnaires included: The patient’s age, spouse’s age, patient’s and spouse’s level of education, patient’s and spouse’s occupation, number of pregnancies (gravida), number of children, duration of marriage, having a private bedroom, and reason for hysterectomy. Sexual Intimacy Questionnaire: This questionnaire was designed based on Bagarozzi’s marital Intimacy Questionnaire^(29)^and was psychometrically evaluated by Shahsiah^(30)^ in Iran. It contains 30 questions and each question is scored on a 4-point Likert scale (always, sometimes, rarely, never). Questions 1 to 4, 12, 13, 14, 16, 20, 22, 27 and 29 are scored in reverse. To determine the reliability of the tool, it was implemented on 140 people and its Cronbach’s alpha coefficient was 0.81.^(30)^

Both online and face-to-face groups followed a question-and-answer (Q&A) and counseling protocol that included eight ninety-minute sessions of positive-approach counseling. To facilitate cooperation and Q&A transactions between the participants and the therapist, the Online Group was divided into three blocks of eleven participants, and the face-to-face group into two blocks of sixteen and seventeen participants. The consent form was sent to the Online Group. Eight sexual therapy group sessions with the intended content (Chart 1) containing educational videos, multimedia, and group discussions were provided and sent to them once a week (agreed on by members) via WhatsApp. They were requested to ask their questions in online chats. Necessary counseling and guidance were provided in the same way by the first researcher. If someone missed a session, its file was provided for them. During therapy, three members could not attend the online sessions. The contents were provided for them, and they asked two questions in a private chat. This group was able to review the sessions because they were recorded.

**Chart 1:** The content of positive approach sessions

Homework	Content	Sessions
Write down positive personal characteristics.	Making an introduction and presenting the rules of the sessions, explaining the purpose of the intervention and treatment logic. Stating the benefits and side-effects of hysterectomy	First
Write down desires, goals, and priorities.	Evaluating pre-therapy changes. Discussing desires, goals, and priorities after hysterectomy. Presenting solution-based questions and techniques. Practicing imagining self in the future and raising hope.	Second
Practice how to apply your capabilities to face problems and track one or several positive traits.	Evaluating client abilities and capabilities with positive practical skills test. Discussing client capabilities and opportunities after hysterectomy. Teaching transfer of learning techniques.	Third
Positive self-monitoring	Performing positive approach, functional analysis, and self-monitoring (write down thoughts and observe positive changes) Performing self-monitoring. Supervising special cases. Asking the miracle question. Mentioning hypothetical solutions. Focusing on clients’ achievements, however small, after hysterectomy.	Fourth
Imagining self in the future	Changing views using previous achievements. Highlighting the positives, helping them find special cases, and using their capabilities and positive traits to face new problems. The cognitive bias changes to positive imagery after hysterectomy.	Fifth
Do a different activity and express personal experiences	Modifying behavior (breaking repetitive problematic behavior patterns, changing action in response to the problem, using contradictions and contrasts, adding new actions to previous patterns in the face of the problem, applying solution-based patterns to solve the problem, taking small steps after hysterectomy)	Sixth
Reading, writing, and burning	Creating positive behaviors, acting differently, noticing its benefits, and observing and collecting evidence aligned with positive thoughts and beliefs. Acting like everything is going to be ok and a miracle has happened (e.g., pretending to have a uterus and fertility is back).	Seventh
Positive writings	Creating a change in feeling based on positive outcomes of hysterectomy like not being worried about getting unintended pregnancies (practicing imagining being in the best situation and expressing feelings. Practicing being in desirable future events in their lives, practicing book of life, practicing positivity) and ultimately summarizing learned skills, identifying potential problems, declaring the end of the sessions.	Eighth

In the face-to-face group, the informed consent form was completed directly. The sessions’ content was similar to that of the online sessions. The face-to-face sessions were held once a week (agreed on by members) in two groups of sixteen and seventeen at Shohadaye Kargar Hospital. One individual could not attend a face-to-face session (Block A) in the second week, so she was directed to a group session in Block B. In addition to their homework being reviewed, in each session, both groups were asked about their sexual intimacy improvements, and they shared their experiences about it based on the contents of the session. Their questions were answered and the next homework was assigned to them if their previous homework had been acceptable. At the beginning of each session, the participants presented the homework assigned at the end of the previous session, and the last fifteen minutes of every session were dedicated to group discussion and Q&A. The main outcome of the study was a change in the average sexual intimacy scores of women after their benign hysterectomy. Sexual intimacy in the online counseling group was assessed with an electronic questionnaire designed by a researcher, and in the face-to-face group, a consent form and a paper sexual intimacy questionnaire were filled. These questionnaires were completed by both groups once at the beginning (baseline), eight weeks after the intervention, and twelve weeks later (flow-up).

After extracting the data from the questionnaire, it was subjected to statistical analysis using SPSS software version 23 (SPSS, Inc., Chicago, IL, USA). Based on the Kolmogorov-Smirnov test, the quantitative variables of marriage duration had a normal distribution, and therefore, in these two cases, the independent parametric t-test was used. Other parameters, including the number of pregnancies and the number of children, did not have a normal distribution, so in these cases, the Mann-Whitney non-parametric test was used. The results of the Kolmogorov-Smirnov test of the sexual intimacy questionnaire were normal all three times (baseline, 8th week, and 12th-week follow-up), so independent parametric t-tests and repeated analysis of variance were used. Satisfactory scores from positive-approach counseling also had a normal distribution, so independent t and paired t parametric tests were used. The significance level of the tests was considered less than 0.05.

This study was approved by the Research Ethics Committee of Shahid Sadoughi University of Medical Sciences, Iran (IR.SSU.REC.1401.080). Before the intervention, oral and written consents were obtained from the participants; all participants were informed of the study goal, method, and the voluntary nature of the research; besides, all were assured of the confidentiality of their information.

## Results

Table 1 shows that the two groups were not significantly different in their qualitative or demographic variables, including occupation (P = 0.31), spouse’s occupation (P = 0.06), education level (P = 0.32), spouse’s education level (P = 0.24), or cause of hysterectomy (P = 0.09). The average age of women was 46.97 ± 2.86 in the online counseling group and 48.3 ± 2.84 in the face-to-face counseling group. These two groups were not different in average age (P = 0.062), number of pregnancies (gravida) (P = 0.88), and number of children (P = 0.71); however, findings showed that the face-to-face group had significantly longer marriages than the online counseling group (P = 0.011) (Table 1).

**Table 1:** Demographic characteristics of participants in two study groups

Variables	Online Group (n = 33)	Face-to-Face Group (n = 33)	p-value
Age (year)	46.97 ± 2.86	48.3 ± 2.84	0.062
Spouse’s age (year)	53.64 ± 3.34	55.24 ± 3.03	0.047
Duration of marriage (year)	23.21 ± 4.03	25.64 ± 3.39	0.011
Gravida	2.73 ± 0.8	2.7 ± 0.88	0.88
Number of children	2.61 ± 0.6	2.55 ± 0.71	0.71
Occupation			0.31
Homemaker	16(48.50)	19(57.60)	
Employee	17(51.10)	14(42.40)	
Spouse’s occupations			0.06
Freelance job	13(39.40)	8(24.20)	
Government clerk	14(42.40)	12(36.40)	
Manual worker	6(18.20)	13(39.40)	
Education level			0.32
High school	5(15.20)	8(24.20)	
High school diploma	9(27.30)	12(36.40)	
Bachelor and higher	19(57.60)	13(39.40)	
Spouse’s education			0.24
High school	3(9.10)	4(12.10)	
High school diploma	13(39.40)	15(36.40)	
Bachelor	11(33.30)	12(36.40)	
Masters’ degree and higher	6(18.20)	2(6.10)	
Cause of hysterectomy			0.09 -
AUB	28(84.80)	32(97)	
Other	5(15.20)	1(3)	

Independent t-test showed that before intervention, the average sexual intimacy score was 72.42 ± 9.05 in the online counseling group which was not significantly higher than the score of 70.21 ± 6.75 in the face-to-face group (P = 0.265). This score was significantly higher in the Online Group after eight weeks (P = 0.001) and at the end of twelve weeks (P = 0.003). Repeated measures ANOVA showed a significant increase in sexual intimacy scores in terms of time (base line. 8th week and 12th week) and the type of intervention (Table 2).

**Table 2:** Comparison of mean sexual intimacy at baseline, 8th week, and flow-up (12th week) after intervention in the two groups

Variables	Online Group (n = 33)	Face-to-Face Group (n = 33)	p-value*
Sexual intimacy score			
Baseline	72.42 ± 9.05	70.21 ± 6.75	0.265*
8 weeks after the intervention	87.06 ± 7.98	81.24 ± 5.55	0.001*
Follow-up (12th week)	90.03 ± 8.23	85.03 ± 5.40	0.003*
P-value**	< 0.001	< 0.001	
	0.043		
P-valued A	< 0.001	< 0.001	
P-valued B	< 0.001	< 0.001	
P-valued C	< 0.001	< 0.001	

* Independent samples t-test; ** Repeated measures; *** Paired t-test; P-valued A: Bonferroni test, for each group between the baseline and week 8; P-valued B: Bonferroni test, for each group between the baseline and week 12; P-valued C: Bonferroni test, for each group between week 8 and week 12

**Figure 2:**
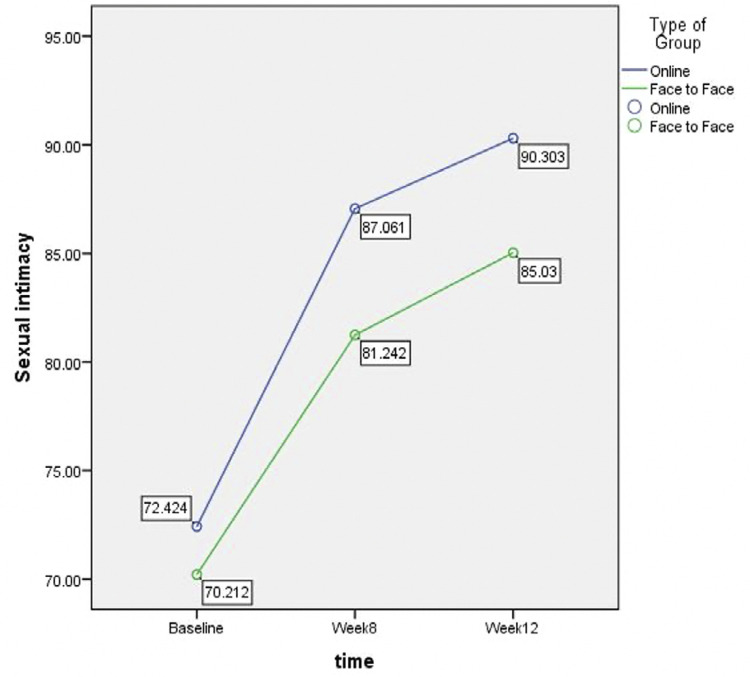
Comparison of the mean sexual intimacy scores of women with hysterectomy

The score of satisfaction with positive-approach counseling in the Online Group after eight weeks and twelve weeks had a significantly better improvement compared with the face-to-face group (P < 0.001). Also, in comparison between the end of the eighth week and the twelfth week, there was a significant increase in satisfaction scores within both groups (P < 0.001) (Figure 2).

In general, it can be concluded that in the present study, the Mean score of sexual intimacy in the online intervention group had a significant increase compared to the face-to-face intervention group at the beginning of the study, the 8th episode and the follow-up time, and also the satisfaction score of the counseling method was also higher in the Online Group than it is significantly more for the Face-to-Face intervention Group. Although both online methods of face-to-face intervention have been able to increase the score of sexual intimacy, considering the type of intervention and the passage of time, it can be concluded that the online method has been more effective in increasing the score of sexual intimacy among couples.

## Discussion

This study compared the effectiveness of online and face-to-face positive-approach counseling on sexual intimacy in women who had undergone benign hysterectomy. The conducted studies indicate that decreased sexual intimacy, satisfaction, and interaction and lack of proper knowledge about how to improve sexual intimacy had led to more marital disputes, mental health deterioration, and decreased sexual intimacy and marital satisfaction.^(15)^ Counseling, educational classes, and a better understanding of sexual relationships were able to improve a couple’s relationship and sexual intimacy.^(31)^ In this study, the average score of sexual intimacy in the online counseling group improved significantly at the end of the eighth and twelfth weeks compared to baseline, which indicated the positive effect of online positive-approach counseling on sexual intimacy in this group. Also, the results of other studies are consistent with our study.^(32)^In these studies, online counseling had a positive effect on the achieved results. Using multimedia software, easy access to content, the anonymity of online therapy participants, and their opportunity to ask questions in the chat make online counseling appealing.

In face-to-face counseling at weeks eight and twelve, there was a significant increase in sexual intimacy scores compared to pre-intervention. The current study results showed that face-to-face positive-approach counseling sessions on sexual and marital relationships improved sexual skills, anxiety, stress, and life satisfaction and led to hope and mental health. Positive-approach interventions identify and elevate positive emotions and improve psychological well-being. They seem to solve problems and disagreements by creating positive behavioral experiences that can improve marital intimacy. Some studies have confirmed the current study results.^(33)^Overall, the results suggest that expressing thoughts and feelings can lead to intimacy in a relationship, which increases sexual intimacy.

Comparing the results of positive-approach counseling in Online and Face-to-Face Groups, although sexual intimacy scores improved in both groups, it was revealed that online counseling improved sexual intimacy more than face-to-face counseling. It can be argued that positive-approach counseling is a suitable counseling technique that, in this study, significantly improved sexual intimacy in both groups. Since the content, length, and frequency of the sessions were similar for the two groups, there was an increase in sexual intimacy scores in both groups, and other studies also indicated that online therapy had been more effective.^(34,35)^The participants in the Face-to-Face Group have to attend many long sessions and travel many times, which makes attendance more difficult for them, leading to lower scores than online counseling scores. The distance of face-to-face sessions could be one of the problems for some patients, which can be solved by holding sessions closer to their residences. Incentives like free sessions could encourage them to attend face-to-face sessions.

Generally, the positive approach of counseling is a non-directive client-centered method that increases clients’ intrinsic motivation for change by identifying and resolving their doubts.

In both groups, the satisfaction score of the counseling method increased significantly at the end of the twelfth week compared to the end of the eighth week. However, the increase in the satisfaction score of online counseling was larger than the increase in the score in face-to-face counseling. The findings of the study showed that the application of online and virtual methods could help evaluate, understand, and treat mental issues in this population.^(36)^

Kamel Ghalibaf et al.^(37)^ also showed that 93.3% of research subjects reported average to high satisfaction with online counseling. Teletherapy provides great flexibility in time and place for the therapist and the client, satisfying the need to meet and solve the problem. Not limited to a certain time or place, online therapy can save time, money, and energy. Online therapy can provide continuity for the rapport formed in person between therapist and client in the clinic, and even when separated by a long distance, protect the client from possible damaging effects of a sudden break in therapy.

According to the findings of other researches, it can be concluded that although traditional face-to-face treatments have advantages such as eye contact and face-to-face communication, online treatment methods due to their simplicity and availability as well as cost reduction compared to face-to-face sessions, provide more convenience for clients.^(15,37)^

Online therapy is similar to face-to-face therapy in content and effectiveness. Still, it has the advantage of being online, making patients more adherent to the therapy and more satisfied with the results. The reason that makes patients more likely to adhere to treatment is anonymity, being comfortable asking questions with less shame, using the experiences and opinions of other participants, not having to travel for face-to-face counseling, and generally, being more satisfied with the results. It can be stated that online positive-approach therapy can help individuals learn how to deal with their problems and assist themselves in case of recurrence.

One of the limitations of this study was Online Group counseling was provided only for women, and it was not possible to provide counseling for their spouses, so women were recommended to make the content of the sessions available to their spouses because intimacy is a marital issue and the husband’s role in developing marital intimacy is crucial. Due to cultural considerations, only women could attend the face-to-face sessions

## Conclusion

Overall, it can be stated that a positive approach to counseling is a suitable method to improve women’s sexual intimacy with a history of hysterectomy. By comparison, online therapy was been more effective than face-to-face therapy maybe because, Iranian women are more interested in online methods as these methods provide better communication with Them culturally due to the privacy of sexual issues. Both Online and Face-to-Face positive counseling can increase the sexual intimacy of post-hysterectomy women and can be recommended for providing care for women with hysterectomy history.
